# Nectin-3 (CD113) Interacts with Nectin-2 (CD112) to Promote Lymphocyte Transendothelial Migration

**DOI:** 10.1371/journal.pone.0077424

**Published:** 2013-10-07

**Authors:** Elisabeth Devilard, Luc Xerri, Patrice Dubreuil, Marc Lopez, Nicolas Reymond

**Affiliations:** 1 Centre de Recherche en Cancérologie de Marseille (CRCM), Aix-Marseille Univ, Marseille, France; 2 Institut Paoli-Calmettes, Marseille, France; 3 INSERM-U1068 (laboratoire hématopoïèse et mécanisme de l’oncogenèse), CNRS-UMR7258, Marseille, France; 4 INSERM-U1068 (laboratoire d’oncologie moléculaire), CNRS-UMR7258, Marseille, France; 5 Centre de Recherche de Biochimie Macromoléculaire (CRBM), CNRS - UMR5237 (laboratoire tyrosine kinases et cancer), Montpellier, France

## Abstract

Lymphocyte trafficking and migration through vascular endothelial cells (ECs) in secondary lymphoid tissues is critical for immune protection. In the present study, we investigate the role of nectin cell adhesion molecules for the migration of lymphocytes through ECs. Nectins are key players for the establishment of homotypic and heterotypic cell to cell contacts; they are required for cell to cell adherens junction formation and take part in the transendothelial migration of monocytes during the step of diapedesis, when monocytes migrate through EC junctions. We first show that Nectin-3 (CD113) is the only nectin expressed by T lymphocytes and since nectins are expressed on ECs we explored Nectin-3 potential functions in lymphocyte: EC interactions. We demonstrate that Nectin-2, expressed on ECs, is the major counter-receptor of Nectin-3. A soluble form of Nectin-3 binds to Nectin-2 localized at EC junctions and blocking Nectin-2 trans-interactions with monoclonal antibodies abolishes the binding of soluble Nectin-3 to ECs. Nectin-2 is expressed on High Endothelial venules (HEVs), where lymphocyte homing occurs *in vivo*. Finally, we show that Nectin-3 trans-interaction with Nectin-2 is essential for the process of lymphocyte transendothelial migration *in vitro* as targeting with blocking monoclonal antibodies either Nectin-3, expressed on lymphocytes, or Nectin-2, expressed on ECs, inhibits lymphocyte extravasation. The nectin family of CAMs is important for the regulation of endothelial barrier functions and transendothelial migration of immune cells. Our results demonstrate for the first time that Nectin-3 trans-interacts with Nectin-2 to promote lymphocyte and monocyte extravasation.

## Introduction

The vascular endothelium consists of a continuous monolayer of cells that lines the entire vascular system. Endothelial junctional complexes are important to control endothelial permeability and create a check-point to regulate the transmigration of large cells such as leukocytes, from the bloodstream to secondary lymphoid organs or underlying tissues [1–7]. Leukocytes leave the bloodstream by interacting with ECs of the vessel walls, through a well-characterized multi-step adhesion molecule cascade [1]. Among the cell adhesion molecules (CAMs) localized at EC junctions [8], PECAM-1 (CD31) [9–11], the JAMs [12–14], CD99 [15], PVR (CD155) [16] and VE-cadherin [17–19] have been described *in vitro* and/or *in vivo* to take part in the leukocyte transmigration process by contributing to the intercellular cohesion and/or by regulating the opening of EC junctions during transmigration. Lymphocyte homing in lymph nodes occurs in high endothelial venules (HEVs) and this process is critical for the homeostatic maintenance of the immune system [20,21]. As the molecular bases that control lymphocyte migration within EC junctions or diapedesis are still largely unknown, the identification of new molecules involved in this process is an important field of research.

Nectins are members of the immunoglobulin superfamily and are structurally related [22]. Four Nectins have been described in humans: Nectin-1 (CD111) [23], Nectin-2 (CD112) [24], Nectin-3 (CD113) [25] and Nectin-4 [26]. They are calcium independent trans-homophilic and trans-heterophilic CAMs [27]. They also trans-interact with the closely related Nectin-like (Necl) molecules and with other less related IgCAMs. We and others previously showed that human Nectin-3 trans-interacts with Nectin-1 and -2 [28] and PVR (also named Necl-5) [26]. Nectin-1 trans-interacts with Nectin-4 [26]. PVR and Nectin-2 also trans-interact with the LFA-1 associated molecule DNAM-1 (CD226) [16]. Nectin associated trans-interactions have been described to ensure natural killer (NK) mediated target cell killing of cancer cells (PVR/DNAM-1 and Nectin-2/DNAM-1) [29] and to regulate monocyte transendothelial migration (PVR/DNAM-1) [16]. Nectins are required for the establishment of adherens cell to cell junctions in epithelial cells [30]. Nectins interact with different scaffold proteins through their cytoplasmic tail including AF-6/Afadin [31] and PICK1 [32] that indirectly link them to the actin cytoskeleton, the E-cadherin system and the JAM family of CAMs.

In the present study, we further investigated the role of Nectins in the trafficking of immune cells and the regulation of EC junctions. We observed that Nectin-3 is the only Nectin expressed on T-lymphocytes, where its function is unknown. As Nectins are expressed on ECs, we explored a possible function of Nectin-3 during the interactions between lymphocytes and ECs.

## Materials and Methods

### Endothelial cells

Primary human umbilical vein endothelial cells (HUVECs) were obtained from Lonza. HUVECs were maintained in EBM2 medium (Lonza) and used before the fifth passage. All cells were cultivated at 37°C in a 5% CO2 atmosphere at constant humidity.

### Isolation of peripheral blood lymphocytes (PBLs)

Peripheral blood mononuclear cells (PBMCs) were purified from cytapheresis (Etablissement Français du Sang Alpes-Méditerranée, Marseille, France). Samples were diluted 1:10 in PBS, layered over Ficoll separation medium (Lymphoprep, Axis-Shield, Norway) and centrifuged at 2000 rpm for 20 min at room temperature (RT) to remove erythrocytes and polymorphonuclear cells (PMNs). PBMCs were collected at the interface then washed twice with PBS. Peripheral Blood Lymphocytes (PBLs) were purified by monocyte depletion after adhesion during four hours in RPMI medium supplemented with 10% fetal calf serum. For each experiment, the percentage of T lymphocytes present in purified PBLs was checked by FACS analysis and was found to be above 75%.

### Antibodies

Monoclonal antibodies (mAbs) against human Nectin-1 (R1.302 (IgG1)), Nectin-2 (R2.477 (IgG1), L14 (IgG2a)), Nectin-3 (N3.2 (IgG2a), N3.12 (IgG2a)), Nectin-4 (N4.40 (IgG1)), PVR (L95 (IgG1)), DNAM-1 (FS123 (IgG1) and KRA236 (IgG2a)) were previously described [16]. Anti-CD99 (Clone hec 2), anti-AF-6 (BD Biosciences), anti-β-catenin (Zymed), anti-CD34 (Immu 133, Beckman Coulter) mAbs and isotypic controls (Beckman Coulter-Immunotech, France) were used. Alexa-488-labeled and TRITC-labeled secondary antibodies were purchased from Molecular Probes (USA).

### Immunohistochemistry

Immuno-detections of Nectin-1, -2, -3 and PVR were performed on frozen sections (5µm) of human placenta, skin and tonsils using different concentrations of mAbs. Specimens were processed with the Universal Dako Kit ChemMate according to the supplier’s recommendations, counter-stained for 5 min in Harris hematoxylin and mounted in Dako glycergel mounting medium.

### Immunofluorescence microscopy

HUVECs were grown on 13-mm round glass coverslips as a confluent monolayer to reach optimal cell to cell contacts. Cells were fixed with 3.7% paraformaldehyde in PBS for 20 min at RT. After blocking aldehydes with 50 mM NH_4_Cl for 10 min at RT, fixed cells were incubated either with 0.2% gelatin (Sigma) in PBS or with 0.2% gelatin and 0.075% saponin (Sigma) in PBS for 20 min. Fixed samples were first labeled with the indicated primary antibodies for 1 h at RT and then incubated with secondary antibodies under the same conditions. Finally, samples were washed, mounted onto slides, embedded with mounting medium (Dako) and visualized using a confocal Leica microscope. Images were processed using the Adobe Photoshop software.

### Production and purification of soluble forms of Nectins

Nectins’ Fc-tagged and DNAM-1 Fc tagged constructions and production have been described previously [26]. Plasmids were purified with endotoxin-free Qiagen kit, sterilized through 0.22 µm filter, aliquoted then stored at -20°C. The different constructions were transfected in COS cells with Fugene ^TM^6 according to the manufacturer recommendations (Promega). Proteins were purified from supernatants on Affi-Gel protein A. Purification was controlled with Coomassie Blue-stained SDS-Page.

### Flow cytometry

Nectins are not sensitive to trypsin treatment. HUVECs were thus trypsinized and resuspended after centrifugation in PBS containing 5% FCS. Cells were then incubated with 2-10 µg/ml of the indicated primary antibodies, then with a secondary phycoerythrin-labeled goat anti-mouse antibody (Immunotech, France). Samples were analyzed using a FACSCalibur and Cell Quest pro (BD Biosciences).

### *In vitro* binding assay

*In vitro* physical interaction studies between Nectins were performed as previously described [26]. Ultrasorb 96-well trays (Nunc) were incubated overnight at 4 °C with 1 µg/ml goat anti-human Fc affinity-purified serum diluted in PBS (Sigma). After three washes with PBS containing 0.5% Tween 20, wells were incubated with PBS containing 1% bovine serum albumin. After three washes, 10^−7^ M of different chimeric Fc proteins (PVR-Fc, Nec1-Fc, Nec2-Fc, Nec3-Fc, and Nec4-Fc) were incubated for 2 h at 37 °C. After the washes, free anti-human-Fc antibodies were blocked with PBS containing 100 µg/ml human immunoglobulin (Novartis) for 1 h at 25 °C. Biotinylated Nec3-Fc (10^−7^ M) was then incubated for 2 h at 37 °C in the absence of Ca^2+^. After three washes, 2 µg/ml streptavidin peroxidase was incubated for 1 h at 37 °C. After five washes, binding was assessed by incubation with the One-Step ABTS (2,2′-azinobis(3-ethylbenzthiazolinesulfonic acid) substrate (Pierce).

### Cell binding assay

HUVECs were trypsinized and resuspended after centrifugation in PBS containing 5% FCS. Cells were either untreated or pre-incubated with 2-10 µg/ml of the indicated antibodies against Nectin-1, Nectin-2, or PVR, alone or in combination for 2 hours at 4 °C. 1.5 µM of different chimeric Fc proteins (Nec3-Fc or DNAM-1-Fc) were incubated for 2 h at 4 °C. After washes, FITC-conjugated goat anti-mouse and PE-conjugated goat anti-human mAbs were then used as secondary reagents and incubated for 1 h at 4 °C. Cells were then analyzed by two color FACS analyses for Nec3-Fc and DNAM-1-Fc binding. Alternatively, HUVECs were transfected with control or 2 different Nectin-2 siRNAs (Invitrogen) using Fugene ^TM^6 according to the manufacturer recommendations (Promega) and cell binding assays were performed three days post transfection.

### Transendothelial migration assay

HUVECs were trypsinized and plated onto 0.1% gelatin (Sigma) coated Costar transwell inserts (5 µm pore size, 6.5-mm diameter), at 3.5 x 10^4^ cells/well. EC monolayers were grown to confluency for four days at 37°C. Permeability of HUVEC monolayers was assessed using FITC-Dextran (38,900 da) at 1 mg/ml. Fluorescence was monitored at 520 nm using a Walla Victor fluorometer. HUVECs were not activated by cytokines. Transmigration experiments with monocytes were performed as described previously [16]. Transmigration experiments with lymphocytes were led by adding 5 x 10^5^ PBLs on each transwell insert. PBLs were let to transmigrate for 3 h at 37°C using SDF1α (250 ng/ml) as a chemo-attractant in the lower chamber. Indicated antibodies were used at a 20 µg/ml concentration. Transmigrated lymphocytes were recovered from the bottom of the well and counted. Cells were finally analyzed by flow cytometry. Each condition was performed in triplicate.

### Statistical analysis

Data are presented as mean + SEM and were analysed using unpaired Student’s *t* test. *P<0.05* was considered statistically significant.

## Results

### Nectin-3 is expressed on T-lymphocytes and binds to EC ligands

**Figure 1:**
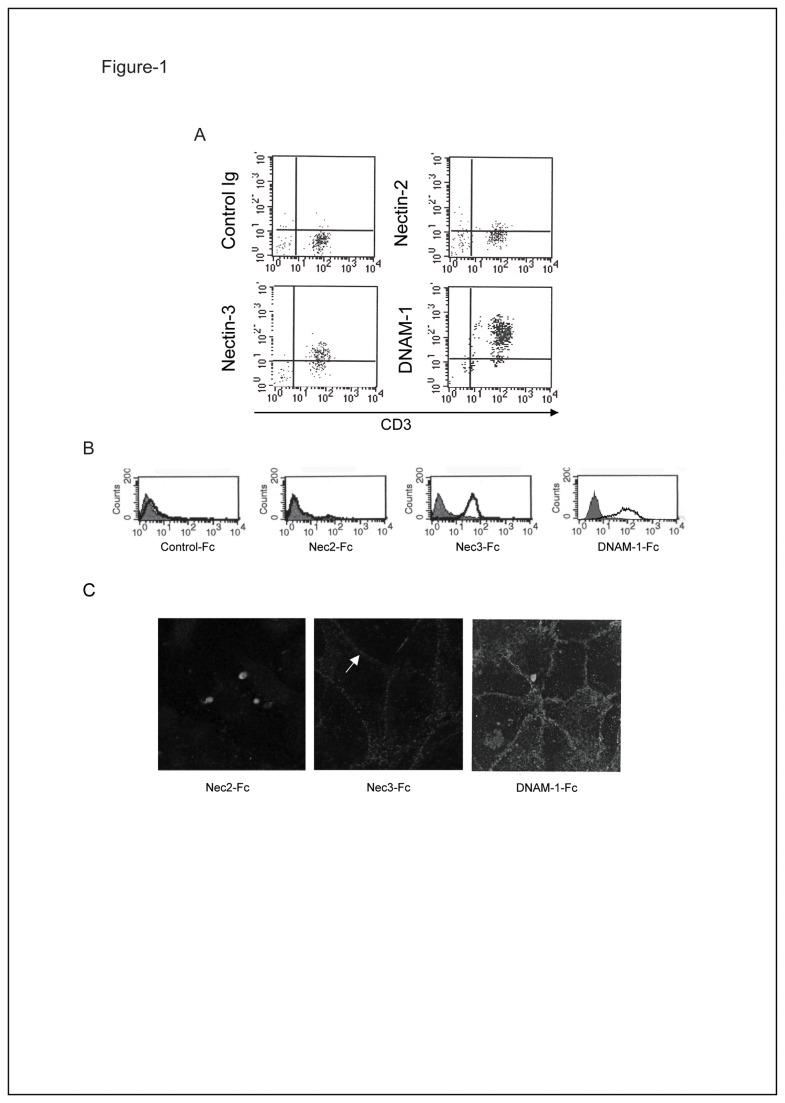
Nectin-3 is expressed on T-lymphocytes and binds to a junctional ligand on ECs. **A**: Fresh PBMCs were gated on lymphocytes on the basis of both size and granularity. Cells were analyzed by two color-immunofluorescence and cytometry with anti-CD3 in combination with anti-Nectin-2 (R2.477), anti-Nectin-3 (N3.12) and anti-DNAM-1 (FS123) mAbs. DNAM-1, previously described to be expressed on T-cells, was taken as a positive control [51]. **B**: Binding of soluble Nectins and DNAM-1 to HUVECs was analyzed by FACS as indicated. **C**: Binding of soluble Nectins and DNAM-1 to HUVECs was analyzed by one color-immunofluorescence as indicated. Bold arrows show junctional stainings. Bar, 50 µm.

Nectins constitute a group of four structurally related CAMs [22]. As we previously reported, PVR (Necl-5) is expressed at EC junctions and interacts with DNAM-1, expressed on monocytes, to regulate monocyte extravasation [16]. To investigate whether Nectins can regulate the extravasation process of other immune cells, we first assessed their expression by FACS analysis on other circulating cells such as lymphocytes. Within the Nectin family, we observed that T-lymphocytes only express Nectin-3 at their cell surface (Figure 1A) and do not express Nectin-1 [33], Nectin-2 (Figure 1A) or Nectin-4 [34].

Nectin-3 is a homophilic and heterophilic CAM [27–29]. We first evaluated whether Nectin-3 counter-receptors are expressed by ECs. We therefore used a recombinant soluble form of Nectin-3 (Nec3-Fc) in a cell binding assay on HUVECs. Soluble Nectin-3 (Nec3-Fc) but not soluble Nectin-2 (Nec2-Fc) bound strongly to ECs, similarly to soluble DNAM-1 (DNAM-1-Fc) providing evidence that some of Nectin-3 ligands are expressed on ECs (Figure 1B). Immunofluorescence analysis of the localization of soluble Nectin-3 binding on ECs revealed that soluble Nectin-3 labeling delineates EC junctions similarly to soluble DNAM-1. Our results therefore suggest that Nectin-3 recognizes ligands expressed at EC junctions (Figure 1C).

### Nectin-3 ligands are expressed at cell to cell junctions and at the apical side of primary ECs

**Figure 2:**
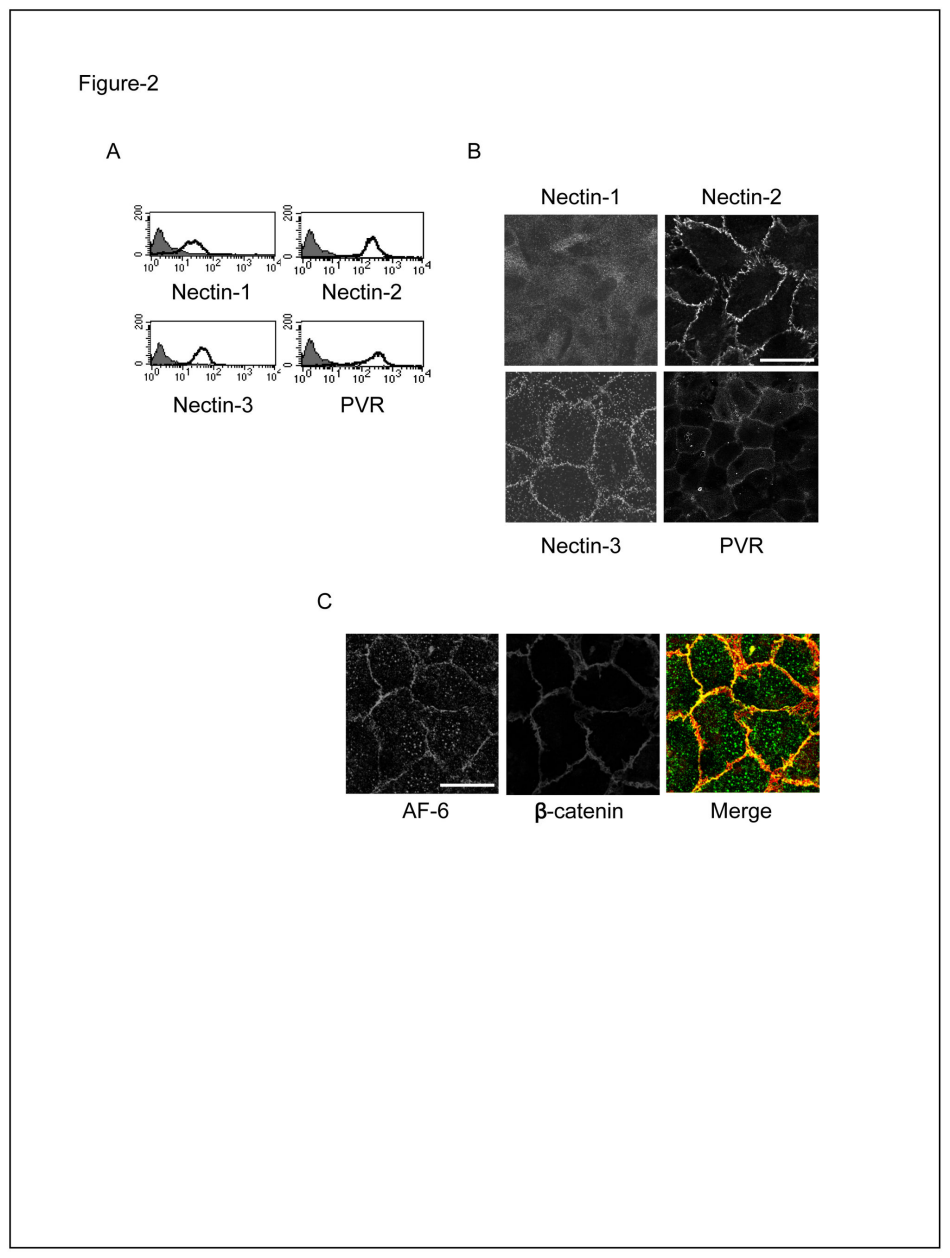
Nectins are expressed on primary ECs. HUVEC confluent monolayers were analyzed by FACS (**A**) and immunofluorescence (**B**) with anti-Nectin-1 (R1.302), anti-Nectin-2 (R2.477), anti-Nectin-3 (N3.12), anti-PVR (PV.404) and (**C**) with anti-AF-6 and anti-β-catenin mAbs. Bar, 50 µm.

According to the Nectin network of trans-interactions, Nectin-3 can trans-interact with itself, Nectin-1, -2 and PVR [27–29]. We initially reported that PVR and Nectin-2 are expressed on vascular ECs such as HUVECs [16]. Using in house anti-Nectins’ mAbs that we previously characterized to be specific, we further investigated the expression of Nectins on primary ECs: FACS analyses showed that Nectin-1 and Nectin-3 are also expressed on HUVECs while Nectin-4 is not (Figure 2A and data not shown). Interestingly, Nectins show different patterns of localization: Nectin-2, -3 and PVR are expressed at EC junctions, as previously described in epithelial cells (Figure 2B) [31]. AF-6, their main cytoplasmic partner, has a highly similar localization as it is also expressed at EC junctions marked by β-catenin (Figure 2C). Nectin-1 is however localized outside EC junctions rather than at intercellular junctions as described in epithelial cells (Figure 1B) [31]. Since soluble Nectin-3 binds to junctional ligands, we can assume that Nectin-1 is not the main counter receptor of Nectin-3 expressed on ECs. Nevertheless, our data show that Nectin-2, -3 and PVR, all expressed at EC junctions, are good candidates to bind to soluble Nectin-3.

### Endothelial Nectin-2 is the main counter-receptor of Nectin-3

**Figure 3:**
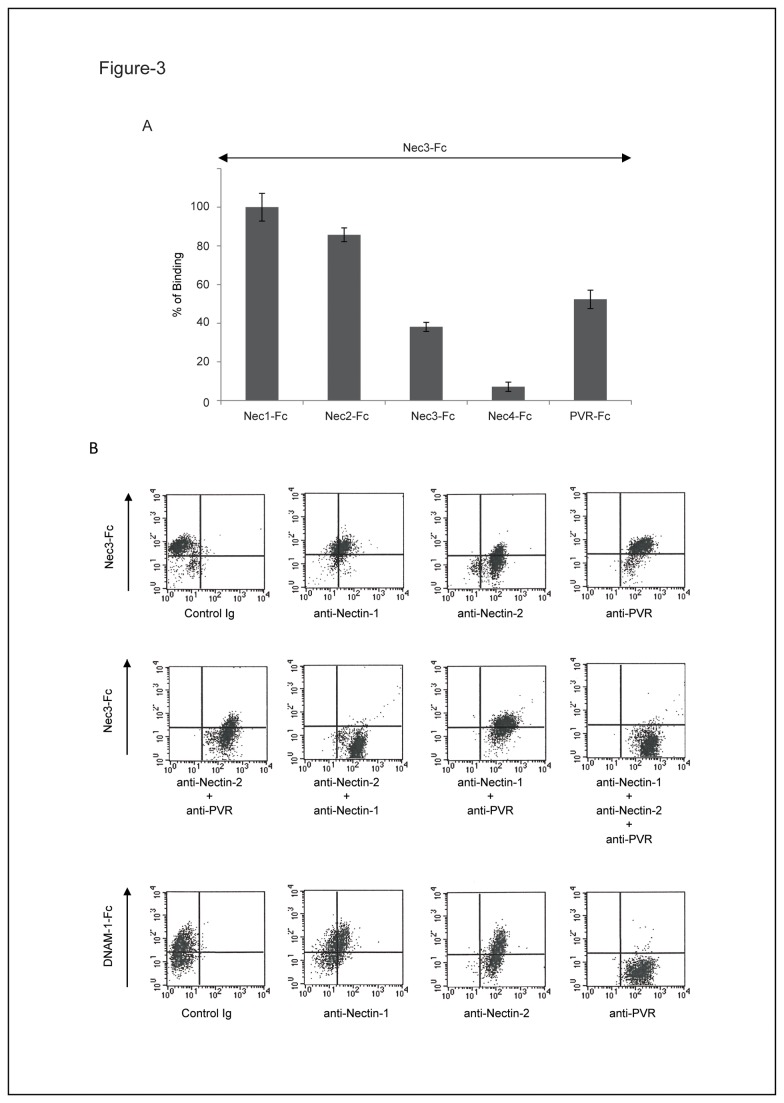
Endothelial Nectin-2 is the major Nectin-3 counter receptor. **A**: Direct binding of soluble Nectin-3 (Nec3-Fc) (10^-7^ M) to indicated immobilized ligands (10^-7^ M). Interactions were measured by ELISA. **B**: Binding of Nec3-Fc (1.5 µM) to HUVECs pre-treated with the indicated mAbs (20 µg/ml). HUVECs were either untreated or pre-treated with anti-Nectin-1 (R1.302), anti-Nectin-2 (R2.477) or anti-PVR (PV.404) mAbs alone or in combinations. Nec3-Fc and DNAM1-Fc binding to HUVECs was then analyzed by FACS.

We then tried to analyze the binding of Nectin-3 to its respective ligands expressed by ECs. We therefore characterized Nectin-3 trans-interactions using our previously described *in vitro* binding assay [26]. We confirm that Nectin-3 can interact with itself (homophilic trans-interactions) and displays three heterophilic direct trans-interactions with Nectin-1, -2 and PVR. Nectin-3 does not bind to Nectin-4. At least *in vitro*, Nectin-3 seems to preferentially bind to Nectin-1 and -2 (Figure 3A). Nectin-3 trans-interacts with PVR, at a level similar to Nectin-3 homophilic trans-interaction (Figure 3A). To discriminate and assess the respective roles of Nectin-1, -2 and PVR on ECs (Figure 3A and B), we tried to block the binding of a soluble form of Nectin-3 (Nec3-Fc) to ECs. Anti-Nectin-1, -2 and anti-PVR mAbs known to functionally block Nectin trans-interactions were used. ECs were double stained and analyzed by FACS to simultaneously monitor the binding of mAbs pre-incubated on ECs and the subsequent binding of Nec3-Fc (Figures 3B and S1A). The binding of Nec3-Fc to ECs was not modulated by anti-PVR mAbs (L95) and only slightly by anti-Nectin-1 mAbs (R1.302). However, anti-Nectin-2 incubation on ECs induced a strong inhibition of Nec3-Fc binding to ECs (Figures 3B and S1A). Interestingly, blocking Nectin-2 and Nectin-1 trans-interactions completely inhibited Nec3-Fc binding to ECs whereas other combinations of mAbs (Nectin-1/PVR or Nectin-2/PVR) were ineffective. As previously described, anti-Nectin-2 mAbs did not modulate soluble DNAM-1 binding to ECs while anti-PVR mAbs did so [16]. Nectin-2 was also depleted from ECs by two different siRNAs and we observed that Nec3-Fc binding was reduced accordingly (Figure S1B). Altogether our results demonstrate that among its ligand expressed on ECs, soluble Nectin-3 preferentially binds to Nectin-2. We therefore confirm that Nectins and their ligands depict some level of specificity as we had previously described that soluble DNAM-1 binds to endothelial PVR [16].

### Nectin-3 functionally interacts with Nectin-2 to promote the transendothelial migration of lymphocytes

**Figure 4:**
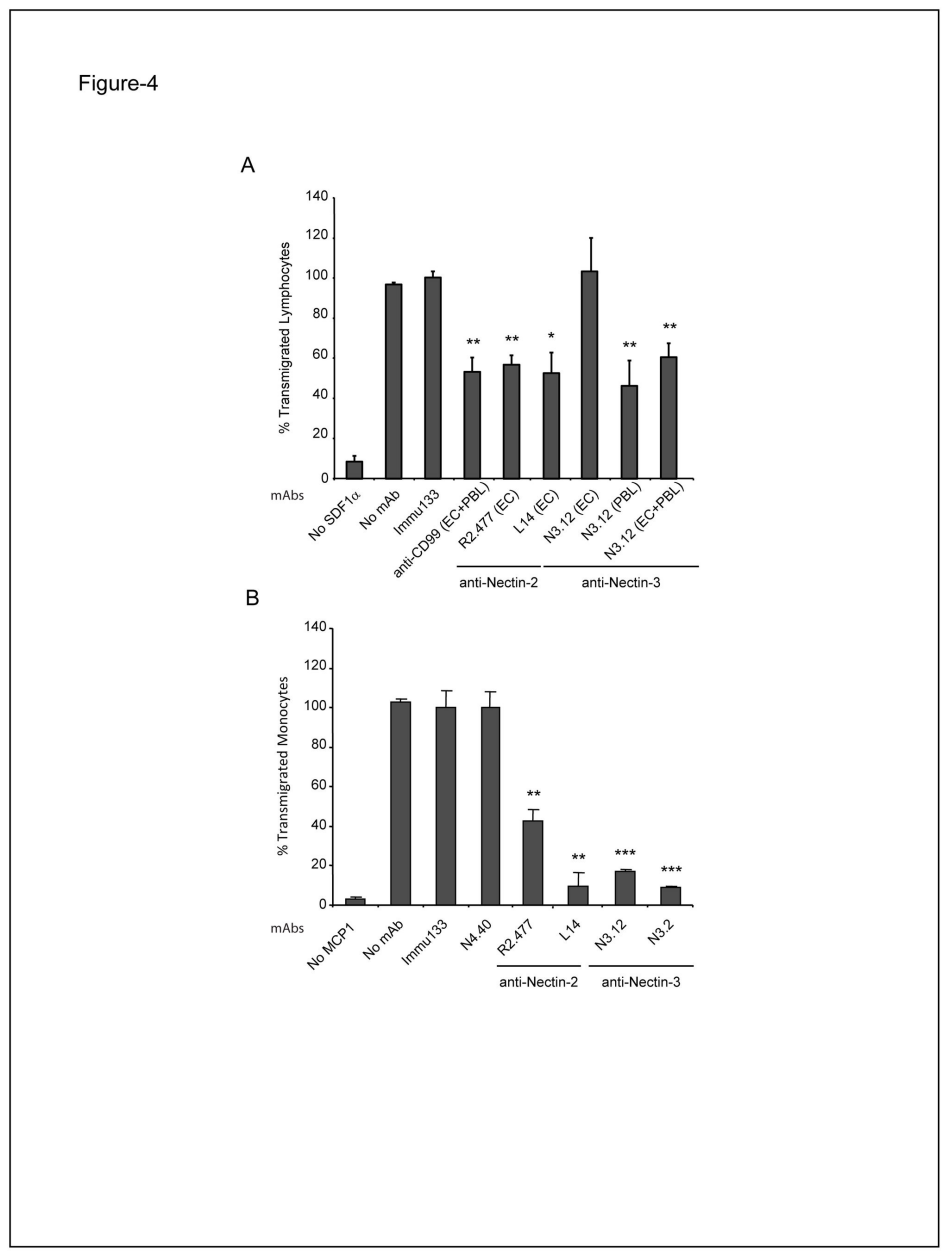
Nectin-2 interacts with Nectin-3 during lymphocyte transmigration. **A**: Lymphocyte transmigration through HUVEC monolayers was performed in the presence of anti-Nectin-2 (R2.477 or L14) blocking mAbs on ECs only, anti-Nectin-3 (N3.12) blocking mAbs on ECs only (EC), lymphocytes only (PBLs) or ECs and lymphocytes (ECs + PBLs). Anti-CD99 blocking mAbs were used as a positive control [35]. **B**: Monocyte transmigration was performed in the presence of anti-Nectin-2 (R2.477 or L.14) or anti-Nectin-3 (N3.12 or N3.2) blocking mAbs. In all experiments, either anti-Nectin-4 (N4.40) or anti-CD34 (Immu133) mAbs were used as isotype matched irrelevant antibodies. The value 100% corresponds to the number of lymphocytes or lymphocytes that transmigrate in the presence of the anti-CD34 mAbs. Each measurement was performed in triplicate. The results were obtained from three independent experiments. Values are mean ± SEM (error bars; N≥3); ***, P < 0.001; **, P < 0.01; *, P < 0.05.

As Nectin-3 is expressed on lymphocytes and soluble Nectin-3 specifically binds to Nectin-2 expressed at EC junctions, we investigated whether the direct trans-interaction between Nectin-3 and -2 plays a role during the lymphocyte extravasation process similarly to the interaction between DNAM-1 and endothelial PVR during monocyte extravasation [16]. We therefore explored the ability of anti-Nectin-2 and anti-Nectin-3 mAbs to block primary lymphocyte transmigration using an *in vitro* transendothelial model based on Boyden chambers. Anti-Nectin-2 (R2.477 or L14) and anti-Nectin-3 (N3.12) blocking mAbs induced a significant inhibition of lymphocyte transmigration when compared to isotype-matched irrelevant antibodies (anti-CD34 mAb) (Figure 4A). Anti-Nectin-2 and -3 mAbs inhibited lymphocyte transmigration similarly to the inhibition obtained after anti-CD99 mAb treatment, taken as a positive control [35]. Importantly, the inhibition of lymphocyte transmigration was observed when anti-Nectin-2 mAbs are incubated only on ECs or when anti-Nectin-3 mAbs are incubated only on lymphocytes (Figure 4A). Also, no change in lymphocyte transmigration was detected when incubating anti-Nectin-3 mAbs only on ECs (Figure 4A). Our results thus identify Nectin-3 on lymphocytes and Nectin-2 on ECs as a new pair of CAMs required for lymphocyte extravasation. Finally, we investigated whether Nectin-3 and -2 specifically regulate lymphocyte transmigration or whether this pair can also promote the extravasation of other circulating immune cells. We found this to be the case since blocking Nectin-3/Nectin-2 binding with mAbs also inhibited monocyte transendothelial migration in a Boyden chamber assay (Figure 4B). This inhibition reaches similar levels to those observed when we block either the Nectin-3/Nectin-2 interaction during lymphocyte extravasation (Figure 4A), or the PVR/DNAM-1 interaction during monocyte transmigration [16]. Altogether, our results demonstrate that Nectin-3 expressed on lymphocytes and monocytes, and endothelial Nectin-2 represent a new pair of transmembrane receptors that functionally trans-interact to promote the transmigration of immune cells.

### Nectin-2 is expressed on High Endothelial Venules

**Figure 5:**
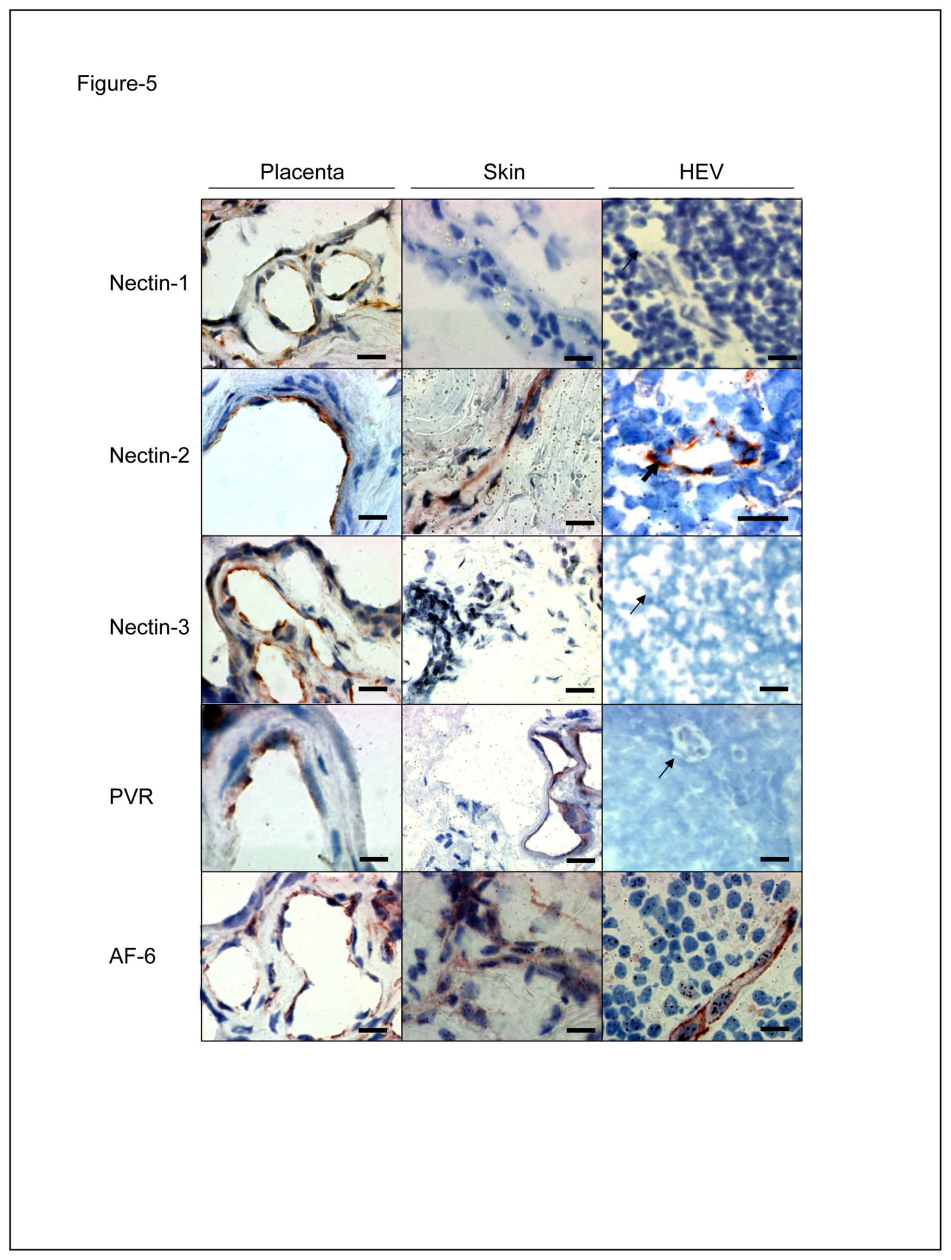
Nectin-2 is expressed on HEVs. Frozen sections of human placenta, skin and lymph nodes were analysed by immunohistochemistry with anti-Nectin-1 (R1.302), anti-Nectin-2 (R2.477), anti-Nectin-3 (N3.12), anti-PVR (PV.404) or anti-AF-6 mAbs. Representative images are shown. Bold arrows show positive specific HEV staining and arrows show negative HEV staining. Bars, 20 µm.

To investigate the functional relevance of the trans-interaction between Nectin-3 and Nectin-2 for the extravasation of lymphocytes, we assessed the expression of Nectins in lymph nodes by immunohistochemistry. We paid particular attention on ECs expressed in these primary tissues i.e. High Endothelial Venules and lymphatic sinuses. Both types of vessels are actively involved in lymphocyte homing *in vivo* [20,21]. Our results show that Nectin-2, but no other Nectins or PVR, is highly expressed on HEVs and on ECs from lymphatic sinuses taken as positive controls (Figure 5, right column). This pattern of expression is specific for lymph nodes: we indeed observed that all Nectins and PVR are expressed on vascular ECs from placental tissue (Figure 5, left column) [16,26]. Nectin-2 and PVR are also expressed on vascular ECs from the skin (Figure 5, middle column). Our results show for the first time that Nectin-2 is the only Nectin expressed *in vivo* on HEVs where it could interact with Nectin-3 expressed on lymphocytes and play a role during lymphocyte extravasation. 

## Discussion

Cell adhesion molecules regulate many physiological processes under normal and pathological conditions. Among them, vascular CAMs regulate the transendothelial migration of circulating cells from blood to secondary lymphoid organs or underlying tissues. They take part in the homing process of lymphocytes, ensuring their patrolling functions, in the extravasation of PMNs and monocytes during the inflammation process, and enable the dissemination of cancer cells from a primary tumour to invade new tissues and form metastases [36].

Identifying new molecules involved in the transmigration process is important to understand its regulation. In the present study, we show that Nectin-3 is a new CAM expressed by lymphocytes. We identify Nectin-2, localized at intercellular junctions, to be the preferential Nectin-3 counter receptor expressed on ECs. We demonstrate that Nectin-3 expressed on lymphocytes functionally trans-interacts with Nectin-2 expressed on ECs to regulate lymphocyte extravasation. As Nectin-2 is highly expressed on HEVs, the Nectin-3/Nectin-2 trans-interaction may therefore be required for an efficient recirculation of lymphocytes throughout the body. Although previous studies have identified endothelial Nectin-2 and PVR to be important for the transmigration of effector memory CD4+ T lymphocytes [37], our results go further as we depict the molecular mechanisms of its implication during lymphocyte extravasation; we identified Nectin-2 expressed on ECs to be Nectin-3 preferential ligand expressed on lymphocytes. As Nectin-3 expression is found on all CD3+ T-cells, we can postulate that both CD4+ and CD8+ T-cell populations may transmigrate through ECs. As we show that Nectin-2 is expressed on HEVs, it will be important in the future to validate our results *in vivo* with mouse models of extravasation and to use ECs from lymph nodes such as HEVs and assess the role of the Nectin-3/Nectin-2 trans-interaction with this model.

Leukocyte extravasation follows a well described CAM cascade [1]. The latest step, referred to as diapedesis, is highly regulated as the permeability of blood vessels is maintained while immune cells migrate through EC junctions. Junctional adhesion molecules perform a guidance/traction function to enable leukocyte migration through EC junctions, as suggested for PECAM-1 [38,39]. The precise step of action of Nectin-2 remains to be explored but due to its localization and similarly to PVR [16], CD99 and PECAM-1 [15], it will mostly be required for the diapedesis step and not during the early adhesion to ECs. PECAM-1, PVR and CD99 act sequentially during the diapedesis step [15,40]. We reported that PVR binds to DNAM-1 to ensure monocyte transmigration [16]. DNAM-1 cis-interacts with LFA-1 and its function is dependent of the expression of LFA-1, as DNAM-1 is not active in Leukocyte Adhesion Deficiency (LAD) patients [41]. As JAM-A is a LFA-1 ligand, we can postulate that CAM complexes may communicate with each other and thus guide leukocytes during the different steps of the diapedesis process. Nectin-2 and JAM-A in ECs may not be cis-associated like LFA-1 and DNAM-1 in leukocytes, but their association could be driven by cytoplasmic scaffold proteins. By analogy with epithelial cells, Nectin-2 on ECs may be connected to JAM-A via their common cytoplasmic partners AF-6 and α-Catenin (Figure 1) [42,43], PICK1 [32] and indirectly to PECAM-1 via Catenins [44,45]. Nectin-2 could also cis-dimerise at cell to cell junctions through its heterophilic interactions and guide the extravasation process. Interestingly, the engagement of Nectins activates different downstream signalling pathways. It leads to the activation of Rac1 and Cdc42, and in parallel it is known that Rac1 activation induces a rapid loss of VE-cadherin adhesion at EC junctions [46]. The trans-interactions between Nectins or PVR expressed on ECs and their ligands expressed on circulating immune cells could therefore induce EC junction opening and promote leukocyte diapedesis during the extravasation process. It will be important to study if Nectins also take part in the trans-cellular route of diapedesis.

Nectin-1 is localized at epithelial adherens junctions and interacts with AF-6 [26,31]. Nectin-3 binds strongly to Nectin-1 *in vitro* using recombinant proteins (Figure 3A) but only slightly on ECs as it seems that Nectin-1 only serves as a Nectin-3 counter receptor when Nectin-2 is not accessible (Figure 3B). Surprisingly, Nectin-1 was not found at EC junctions but was expressed diffusely, possibly at the apical part of ECs (Figure 1B). Similar disparity has been described for members of the Cadherin family: VE-cadherin is expressed at EC junctions while N-cadherin is expressed at the apical part of ECs. A dominant signal localized in the cytoplasmic tail of VE-cadherin prevents N-cadherin localization to EC junctions [47,48]. Nectin-1, while excluded from cell to cell junctions, could thus play a different role during the extravasation process of circulating cells. Our results demonstrate that Nectins, even when they are expressed in the same tissues or on the same cells, could display different specificity of trans-interactions and thereby carry out different functions.

## Conclusions

We present here, the first demonstration that Nectin-3 and -2 form a new pair of receptors that promotes lymphocyte and monocyte transendothelial migration. Some CAMs required for leukocyte extravasation can also play a role during cancer cell extravasation and the formation of metastases [49,50]. As Nectins are highly expressed in cancer cells, it will be important to investigate whether they are implicated in cancer cell extravasation as well as in inflammatory diseases. 

## Supporting Information

Figure S1 **Nectin-3 major counter receptor on ECs is Nectin-2.** **A**: Complementary bar graph representing the binding of Nec3-Fc and DNAM-1-Fc (1.5 µM) to HUVECs treated with the indicated mAbs as shown in Figure 3B. **B**: HUVECs were transfected with the indicated siRNAs. A representative histogram of Nectin-2 cell surface expression by FACS analysis is shown (left panel). Nec3-Fc binding (1.5 µM) to HUVECs after siRNA transfection of HUVECs with the indicated siRNAs is presented (right panel).
(TIF)

## References

[1] Springer TA (1994) Traffic signals for lymphocyte recirculation and leukocyte emigration: the multistep paradigm. Cell 76: 301-314. doi:10.1016/0092-8674(94)90337-9. PubMed: 7507411. 7507411

[2] Luscinskas FW, Ma S, Nusrat A, Parkos CA, Shaw SK (2002) Leukocyte transendothelial migration: a junctional affair. Semin Immunol 14: 105-113. doi:10.1006/smim.2001.0347. PubMed: 11978082. 11978082

[3] Worthylake RA, Burridge K (2001) Leukocyte transendothelial migration: orchestrating the underlying molecular machinery. Curr Opin Cell Biol 13: 569-577. doi:10.1016/S0955-0674(00)00253-2. PubMed: 11544025. 11544025

[4] Johnson-Léger C, Aurrand-Lions M, Imhof BA (2000) The parting of the endothelium: miracle, or simply a junctional affair? J Cell Sci 113(6): 921-933. PubMed: 10683141. 10683141 10.1242/jcs.113.6.921

[5] Weber C (2003) Novel mechanistic concepts for the control of leukocyte transmigration: specialization of integrins, chemokines, and junctional molecules. J Mol Med (Berl) 81: 4-19. PubMed: 12545245. 12545245 10.1007/s00109-002-0391-x

[6] Vestweber D (2012) Relevance of endothelial junctions in leukocyte extravasation and vascular permeability. Ann N Y Acad Sci 1257: 184-192. doi:10.1111/j.1749-6632.2012.06558.x. PubMed: 22671605. 22671605

[7] Vestweber D (2012) Novel insights into leukocyte extravasation. Curr Opin Hematol 19: 212-217. doi:10.1097/MOH.0b013e3283523e78. PubMed: 22395664. 22395664

[8] Dejana E (2004) Endothelial cell-cell junctions: happy together. Nat Rev Mol Cell Biol 5: 261-270. doi:10.1038/nrm1357. PubMed: 15071551. 15071551

[9] Muller WA, Weigl SA, Deng X, Phillips DM (1993) PECAM-1 is required for transendothelial migration of leukocytes. J Exp Med 178: 449-460. doi:10.1084/jem.178.2.449. PubMed: 8340753. 8340753 PMC2191108

[10] Liao F, Ali J, Greene T, Muller WA (1997) Soluble domain 1 of platelet-endothelial cell adhesion molecule (PECAM) is sufficient to block transendothelial migration in vitro and in vivo. J Exp Med 185: 1349-1357. doi:10.1084/jem.185.7.1349. PubMed: 9104821. 9104821 PMC2196259

[11] Bogen S, Pak J, Garifallou M, Deng X, Muller WA (1994) Monoclonal antibody to murine PECAM-1 (CD31) blocks acute inflammation in vivo. J Exp Med 179: 1059-1064. doi:10.1084/jem.179.3.1059. PubMed: 8113674. 8113674 PMC2191427

[12] Martìn-Padura I, Lostaglio S, Schneemann M, Williams L, Romano M et al. (1998) Junctional adhesion molecule, a novel member of the immunoglobulin superfamily that distributes at intercellular junctions and modulates monocyte transmigration. J Cell Biol 142: 117-127. doi:10.1083/jcb.142.1.117. PubMed: 9660867. 9660867 PMC2133024

[13] Del Maschio A, De Luigi A, Martin-Padura I, Brockhaus M, Bartfai T et al. (1999) Leukocyte recruitment in the cerebrospinal fluid of mice with experimental meningitis is inhibited by an antibody to junctional adhesion molecule (JAM). J Exp Med 190: 1351-1356. doi:10.1084/jem.190.9.1351. PubMed: 10544206. 10544206 PMC2195675

[14] Ostermann G, Weber KS, Zernecke A, Schröder A, Weber C (2002) JAM-1 is a ligand of the beta(2) integrin LFA-1 involved in transendothelial migration of leukocytes. Nat Immunol 3: 151-158. doi:10.1038/nrm770. PubMed: 11812992. 11812992

[15] Schenkel AR, Mamdouh Z, Chen X, Liebman RM, Muller WA (2002) CD99 plays a major role in the migration of monocytes through endothelial junctions. Nat Immunol 3: 143-150. doi:10.1038/ni749. PubMed: 11812991. 11812991

[16] Reymond N, Imbert AM, Devilard E, Fabre S, Chabannon C et al. (2004) DNAM-1 and PVR regulate monocyte migration through endothelial junctions. J Exp Med 199: 1331-1341. doi:10.1084/jem.20032206. PubMed: 15136589. 15136589 PMC2211807

[17] Dejana E, Bazzoni G, Lampugnani MG (1999) Vascular endothelial (VE)-cadherin: only an intercellular glue? Exp Cell Res 252: 13-19. doi:10.1006/excr.1999.4601. PubMed: 10502395. 10502395

[18] Broermann A, Winderlich M, Block H, Frye M, Rossaint J et al. (2011) Dissociation of VE-PTP from VE-cadherin is required for leukocyte extravasation and for VEGF-induced vascular permeability in vivo. J Exp Med 208: 2393-2401. doi:10.1084/jem.20110525. PubMed: 22025303. 22025303 PMC3256962

[19] Schulte D, Küppers V, Dartsch N, Broermann A, Li H et al. (2011) Stabilizing the VE-cadherin-catenin complex blocks leukocyte extravasation and vascular permeability. EMBO J 30: 4157-4170. doi:10.1038/emboj.2011.304. PubMed: 21857650. 21857650 PMC3199392

[20] Bai Z, Cai L, Umemoto E, Takeda A, Tohya K et al. (2013) Constitutive Lymphocyte Transmigration across the Basal Lamina of High Endothelial Venules Is Regulated by the Autotaxin/Lysophosphatidic Acid Axis. J Immunol, 190: 2036–48. PubMed: 23365076. 23365076 10.4049/jimmunol.1202025

[21] Umemoto E, Hayasaka H, Bai Z, Cai L, Yonekura S et al. (2011) Novel regulators of lymphocyte trafficking across high endothelial venules. Crit Rev Immunol 31: 147-169. doi:10.1615/CritRevImmunol.v31.i2.40. PubMed: 21542791. 21542791

[22] Takai Y, Miyoshi J, Ikeda W, Ogita H (2008) Nectins and nectin-like molecules: roles in contact inhibition of cell movement and proliferation. Nat Rev Mol Cell Biol 9: 603-615. doi:10.1038/nrm2457. PubMed: 18648374. 18648374

[23] Lopez M, Eberlé F, Mattei MG, Gabert J, Birg F et al. (1995) Complementary DNA characterization and chromosomal localization of a human gene related to the poliovirus receptor-encoding gene. Gene 155: 261-265. doi:10.1016/0378-1119(94)00842-G. PubMed: 7721102. 7721102

[24] Eberlé F, Dubreuil P, Mattei MG, Devilard E, Lopez M (1995) The human PRR2 gene, related to the human poliovirus receptor gene (PVR), is the true homolog of the murine MPH gene. Gene 159: 267-272. doi:10.1016/0378-1119(95)00180-E. PubMed: 7622062. 7622062

[25] Reymond N, Borg JP, Lecocq E, Adelaide J, Campadelli-Fiume G et al. (2000) Human nectin3/PRR3: a novel member of the PVR/PRR/nectin family that interacts with afadin. Gene 255: 347-355. doi:10.1016/S0378-1119(00)00316-4. PubMed: 11024295. 11024295

[26] Reymond N, Fabre S, Lecocq E, Adelaïde J, Dubreuil P et al. (2001) Nectin4/PRR4, a new afadin-associated member of the nectin family that trans-interacts with nectin1/PRR1 through V domain interaction. J Biol Chem 276: 43205-43215. doi:10.1074/jbc.M103810200. PubMed: 11544254. 11544254

[27] Lopez M, Aoubala M, Jordier F, Isnardon D, Gomez S et al. (1998) The human poliovirus receptor related 2 protein is a new hematopoietic/endothelial homophilic adhesion molecule. Blood 92: 4602-4611. PubMed: 9845526. 9845526

[28] Satoh-Horikawa K, Nakanishi H, Takahashi K, Miyahara M, Nishimura M et al. (2000) Nectin-3, a new member of immunoglobulin-like cell adhesion molecules that shows homophilic and heterophilic cell-cell adhesion activities. J Biol Chem 275: 10291-10299. doi:10.1074/jbc.275.14.10291. PubMed: 10744716. 10744716

[29] Bottino C, Castriconi R, Pende D, Rivera P, Nanni M et al. (2003) Identification of PVR (CD155) and Nectin-2 (CD112) as cell surface ligands for the human DNAM-1 (CD226) activating molecule. J Exp Med 198: 557-567. doi:10.1084/jem.20030788. PubMed: 12913096. 12913096 PMC2194180

[30] Takai Y, Ikeda W, Ogita H, Rikitake Y (2008) The immunoglobulin-like cell adhesion molecule nectin and its associated protein afadin. Annu Rev Cell Dev Biol 24: 309-342. doi:10.1146/annurev.cellbio.24.110707.175339. PubMed: 18593353. 18593353

[31] Takahashi K, Nakanishi H, Miyahara M, Mandai K, Satoh K et al. (1999) Nectin/PRR: an immunoglobulin-like cell adhesion molecule recruited to cadherin-based adherens junctions through interaction with Afadin, a PDZ domain-containing protein. J Cell Biol 145: 539-549. doi:10.1083/jcb.145.3.539. PubMed: 10225955. 10225955 PMC2185068

[32] Reymond N, Garrido-Urbani S, Borg JP, Dubreuil P, Lopez M (2005) PICK-1: a scaffold protein that interacts with Nectins and JAMs at cell junctions. FEBS Lett 579: 2243-2249. doi:10.1016/j.febslet.2005.03.010. PubMed: 15811349. 15811349

[33] Lopez M, Jordier F, Bardin F, Coulombel L, Chabannon C et al. (1997) Leukocyte TypingVI. White Cell Differ Antigens: 1081-1083.

[34] Fabre-Lafay S, Monville F, Garrido-Urbani S, Berruyer-Pouyet C, Ginestier C et al. (2007) Nectin-4 is a new histological and serological tumor associated marker for breast cancer. BMC Cancer 7: 73. doi:10.1186/1471-2407-7-73. PubMed: 17474988. 17474988 PMC1868744

[35] Bixel G, Kloep S, Butz S, Petri B, Engelhardt B et al. (2004) Mouse CD99 participates in T-cell recruitment into inflamed skin. Blood 104: 3205-3213. doi:10.1182/blood-2004-03-1184. PubMed: 15280198. 15280198

[36] Madsen CD, Sahai E (2010) Cancer dissemination--lessons from leukocytes. Dev Cell 19: 13-26. doi:10.1016/j.devcel.2010.06.013. PubMed: 20643347. 20643347

[37] Manes TD, Pober JS (2011) Identification of endothelial cell junctional proteins and lymphocyte receptors involved in transendothelial migration of human effector memory CD4+ T cells. J Immunol 186: 1763-1768. doi:10.4049/jimmunol.1002835. PubMed: 21191062. 21191062 PMC3417063

[38] Mamdouh Z, Chen X, Pierini LM, Maxfield FR, Muller WA (2003) Targeted recycling of PECAM from endothelial surface-connected compartments during diapedesis. Nature 421: 748-753. doi:10.1038/nature01300. PubMed: 12610627. 12610627

[39] Muller WA (2003) Leukocyte-endothelial-cell interactions in leukocyte transmigration and the inflammatory response. Trends Immunol 24: 327-334. PubMed: 12810109. 12810109 10.1016/s1471-4906(03)00117-0

[40] Sullivan DP, Seidman MA, Muller WA (2013) Poliovirus receptor (CD155) regulates a step in transendothelial migration between PECAM and CD99. Am J Pathol 182: 1031-1042. doi:10.1016/j.ajpath.2012.11.037. PubMed: 23333754. 23333754 PMC3586692

[41] Shibuya K, Lanier LL, Phillips JH, Ochs HD, Shimizu K et al. (1999) Physical and functional association of LFA-1 with DNAM-1 adhesion molecule. Immunity 11: 615-623. doi:10.1016/S1074-7613(00)80136-3. PubMed: 10591186. 10591186

[42] Ebnet K, Suzuki A, Horikoshi Y, Hirose T, Meyer Zu Brickwedde MK et al. (2001) The cell polarity protein ASIP/PAR-3 directly associates with junctional adhesion molecule (JAM). EMBO J 20: 3738-3748. doi:10.1093/emboj/20.14.3738. PubMed: 11447115. 11447115 PMC125258

[43] Itoh M, Sasaki H, Furuse M, Ozaki H, Kita T et al. (2001) Junctional adhesion molecule (JAM) binds to PAR-3: a possible mechanism for the recruitment of PAR-3 to tight junctions. J Cell Biol 154: 491-497. doi:10.1083/jcb.200103047. PubMed: 11489913. 11489913 PMC2196413

[44] Biswas P, Zhang J, Schoenfeld JD, Schoenfeld D, Gratzinger D et al. (2005) Identification of the regions of PECAM-1 involved in beta- and gamma-catenin associations. Biochem Biophys Res Commun 329: 1225-1233. doi:10.1016/j.bbrc.2005.02.095. PubMed: 15766557. 15766557

[45] Ilan N, Mahooti S, Rimm DL, Madri JA (1999) PECAM-1 (CD31) functions as a reservoir for and a modulator of tyrosine-phosphorylated beta-catenin. J Cell Sci 112 18: 3005-3014. PubMed: 10462517. 10462517 10.1242/jcs.112.18.3005

[46] van Buul JD, Anthony EC, Fernandez-Borja M, Burridge K, Hordijk PL (2005) Proline-rich tyrosine kinase 2 (Pyk2) mediates vascular endothelial-cadherin-based cell-cell adhesion by regulating beta-catenin tyrosine phosphorylation. J Biol Chem 280: 21129-21136. doi:10.1074/jbc.M500898200. PubMed: 15778498. 15778498

[47] Giampietro C, Taddei A, Corada M, Sarra-Ferraris GM, Alcalay M et al. (2012) Overlapping and divergent signaling pathways of N-cadherin and VE-cadherin in endothelial cells. Blood 119: 2159-2170. doi:10.1182/blood-2011-09-381012. PubMed: 22246030. 22246030

[48] Navarro P, Ruco L, Dejana E (1998) Differential localization of VE- and N-cadherins in human endothelial cells: VE-cadherin competes with N-cadherin for junctional localization. J Cell Biol 140: 1475-1484. doi:10.1083/jcb.140.6.1475. PubMed: 9508779. 9508779 PMC2132661

[49] Ghislin S, Obino D, Middendorp S, Boggetto N, Alcaide-Loridan C et al. (2011) Junctional adhesion molecules are required for melanoma cell lines transendothelial migration in vitro. Pigment Cell. Melanoma Res 24: 504-511. 10.1111/j.1755-148X.2011.00856.x 21466663

[50] Reymond N, Im JH, Garg R, Vega FM, d’Agua Borda B, et al. (2012). p. Cd c42 promotes transendothelial migration of cancer cells through beta1 integrin. J Cell Biol 199: 653-668

[51] Chan CJ, Andrews DM, Smyth MJ (2012) Receptors that interact with nectin and nectin-like proteins in the immunosurveillance and immunotherapy of cancer. Curr Opin Immunol 24: 246-251. doi:10.1016/j.coi.2012.01.009. PubMed: 22285893. 22285893

