# Supplementary figures and images for: Nectin-3 (CD113) Interacts with Nectin-2 (CD112) to Promote Lymphocyte Transendothelial Migration
Source: PLoS One. 2013 Oct 7;8(10):e77424. doi: 10.1371/journal.pone.0077424 (PMC3792040; doi:10.1371/journal.pone.0077424)

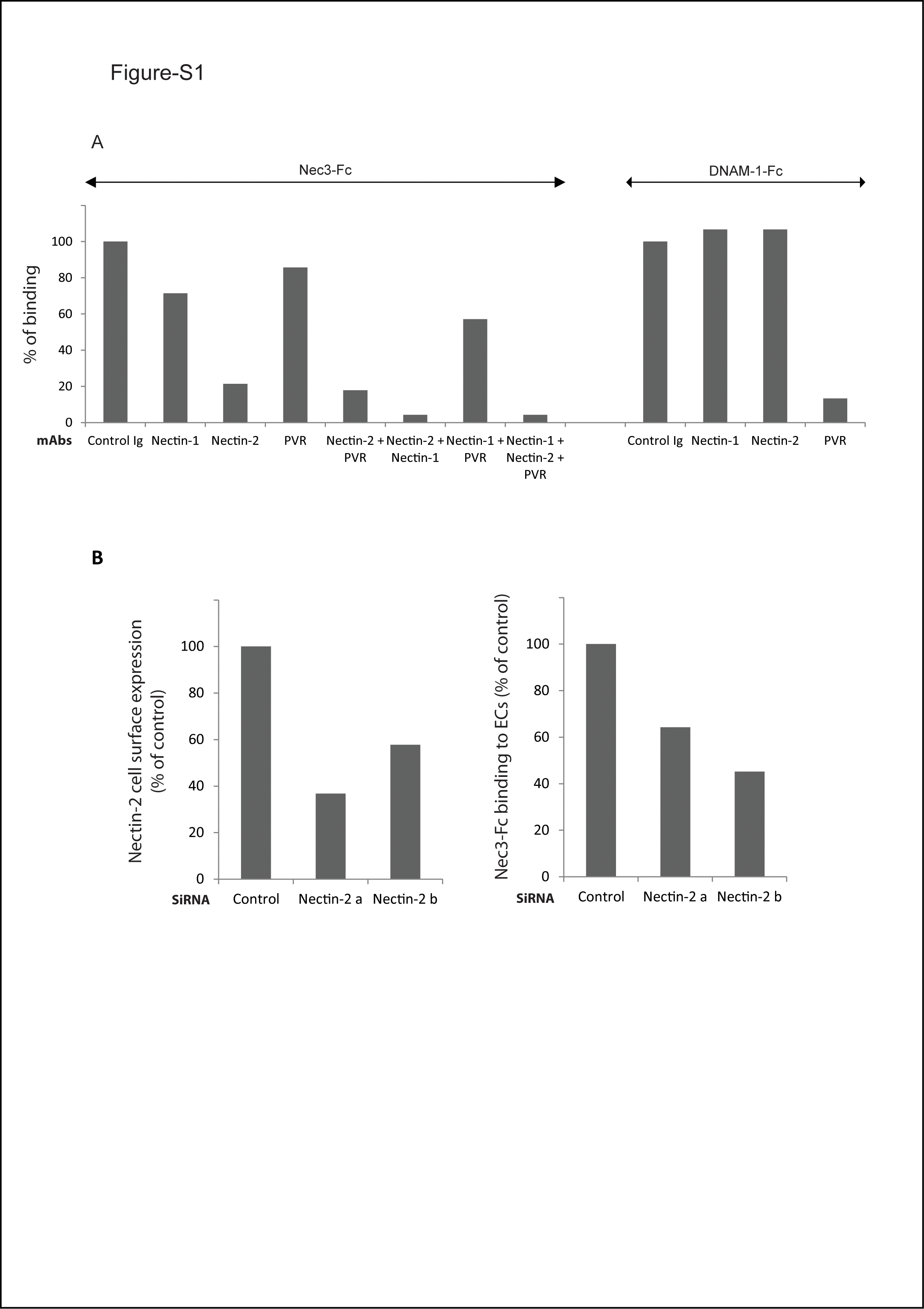

Supplement: Figure S1 — Nectin-3 major counter receptor on ECs is Nectin-2. A: Complementary bar graph representing the binding of Nec3-Fc and DNAM-1-Fc (1.5 µM) to HUVECs treated with the indicated mAbs as shown in Figure 3B. B: HUVECs were transfected with the indicated siRNAs. A representative histogram of Nectin-2 cell surface expression by FACS analysis is shown (left panel). Nec3-Fc binding (1.5 µM) to HUVECs after siRNA transfection of HUVECs with the indicated siRNAs is presented (right panel). (TIF) [file pone.0077424.s001.tif]
